# *Dunno if you've any plans for the future*: medical student indirect questioning in simulated oncology interviews

**DOI:** 10.1186/1472-6920-12-8

**Published:** 2012-03-02

**Authors:** Céline Bourquin, Friedrich Stiefel, Alexandre Berney, Pascal Singy

**Affiliations:** 1 Psychiatric Liaison Service, Lausanne University Hospital, Les Allières, 1011 Lausanne-CHUV, Switzerland

## Abstract

**Background:**

This exploratory study investigated the motives of medical students (N = 63) for using indirect questions of the type *I don't know if *[you have already heard about chemotherapies], *I don't know how *[you are], or *I don't know what *[you do for a living] in simulated patient interviews during a communication skills course.

**Methods:**

*I don't know *questions (IDK-Qs) were observed during the initial evaluation of students' communication skills; they were systematically identified through video screening and subjected to a qualitative content and discourse analysis considering their context, their content, their intent and their effect on the simulated patients. To evaluate the specificity of medical students' IDK-Qs, the data were compared with a data set of oncologists (N = 31) conducting simulated patient interviews in the context of a Communication Skills Training (CST).

**Results:**

During the interviews, 41.3% of the students asked 1-6 IDK-Qs. The IDK-Qs were attributed to three content categories: medical/treatment questions (N = 24); lifestyle/psychosocial questions (N = 18); and "inviting questions" questions (N = 11). Most of the IDK-Qs had an exploratory function (46/53), with simulated patients providing detailed responses or asking for more information (36/53). IDK-Qs were rare in the oncologist sample compared to the student sample (5 vs. 53 occurrences).

**Conclusions:**

IDK-Qs showed a question design difference between medical students and oncologists in simulated patient interviews. Among other reasons for this difference, the possible function of IDK-Qs as a protective linguistic strategy and marker for psychological discomfort is discussed.

## Background

The practice of breaking bad news in oncology has been investigated with different methods and from different point of views, focusing on communication style (patient-centered vs. emotion- or disease-centered) [1-7], skills (tailoring the information to the patient's needs, involving him/her in decision-making, disclosing concerns, etc.) [2,3,8-15] and communication skills training programs (CST) at the under- and postgraduate level [16-30].

With regard to the required communication skills for breaking bad news, even though the focus has also been on *how *things are said [9-12,14] - by identifying communication practices concerning most notably how physicians respond to cancer patients' problems and concerns - it has rather emphasized *what kind *of information is conveyed or gathered [4,6-8,13,15]. This exploratory study examined in a simulated oncology setting assertions used as questions of the type *I don't know if *[you have already heard about chemotherapies], *I don't know how *[you are], or *I don't know what *[you do for a living], which are a conventional indirect formulation of the question. The focus was hence on *how *information is gathered.

The topic of question design in physician-patient interaction has been specifically addressed in the early work of Mishler [31], which aimed at an understanding of how the discursive work of clinical medicine is done, and of Cassel [32], which detailed formal aspects of the verbal exchanges between physicians and their patients; and in the more recent work of Boyd and Heritage [33], examining principles underlying the question design in past and personal history-taking, and of Roter and Hall [34], focusing on implications of physicians' closed- vs. open-ended questions in medical visits. Based in primary care settings and observed naturally occurring interactions, these studies provide significant input regarding the analysis of question design in medical communication. Still, to the best of our knowledge, the *I don't know *question design has not yet been examined in any medical interviews with actual or simulated patients.

The study was based on data of a project assessing a course at the University of Lausanne for medical students on breaking bad news. The *I don't know questions *(IDK-Qs) were observed during the initial assessment of students' communication skills using the RIAS [35,36]. Their recurrence in the students' discourse led to an exploratory study with the aim to identify (i) the discursive contents (biomedical, psychosocial, etc.) of IDK-Qs, (ii) their intents (specific, probing or exploratory) and (iii) their effects on the (simulated) patients (blocking or inviting verbal expression).

It was hypothesized that psychological as well as sociolinguistic factors explain the recurrence of IDK-Qs in the students' discourse. From a psychological point of view, the students' IDK-Qs could represent, for example, a strategy for social distancing or avoidance and thus a marker for insecurity or emotional protection. It has already been investigated how psychological processes, mediated by defense mechanisms, can protect against emotional distress associated with (simulated) patient interviews [37-39]. From a sociolinguistic point of view, IDK-Qs could be, for example, a generational and/or a sociolectal marker or a strategy to minimize the imposition of a speech act regarded as face-threatening (for the student or the simulated patient); a speech act may, for example, threaten face by impeding freedom of action or by potentially indicating that the other's feelings, concerns and wants do not matter [40].

## Methods

### Subjects and material

The material consisted of videotaped interviews with a simulated patient (lasting about 20 min), conducted by 63 fourth year (first year of Master's degree) medical students. The interviews were based on two scenarios: disclosure of breast cancer for which adjuvant chemotherapy and radiotherapy with curative intent are proposed or disclosure of stomach cancer for which chemotherapy with palliative intent is proposed.

To assess the specificity of students' IDK-Qs, the data were compared with a pre-existing data set of oncologists (N = 31) conducting videotaped interviews with a simulated patient based on the same two scenarios in the context of a CST [37-39].

The student and the oncologist groups were comparable with regard to male/female distribution, with 60% (N = 38) of female students and 65% (N = 20) of female oncologists, and scenario distribution, with 33 interviews with the curative and 30 with the palliative scenario in the student group and 15 interviews with the curative and 16 with the palliative scenario in the oncologist group.

The mean length of clinical experience for oncologists was 12 years (SD = 7.5).

### Student and oncologist training in communication skills

At the Faculty of Biology and Medicine of the Lausanne University, the teaching of physician-patient communication for medical students has, for more than the last 10 years, consisted in the first teaching year of an eight-hour introduction (plenary sessions), followed in the second year by analyses of videotaped consultations and role-plays in small groups (two 2-hour sessions) and plenary lectures (6 hours), in the third year by supervised interviews with patients in small groups (six 2-hour sessions), and in the fourth year by an introduction to breaking bad news based on videotaped interviews of students with simulated patients in small groups (two 2-hour sessions).

The CST for oncologists consists of a 2-day course mainly based on interactivity and practical exercises, e.g. role-plays, critical incident reports, video-analyses of participants' interviews with a simulated patient, followed by individual supervisions and a 1-day follow-up course. In Switzerland, CST became mandatory in 2005 for physicians specializing in oncology. The oncologists whose simulated patient interviews were analyzed in this study had no previous training in communication skills.

### Data analysis

IDK-Qs were systematically identified through video screening and transcribed, together with the previous and next related utterances (i.e., simulated patient responses, re-statements, repetitions, etc.).

The data were subjected to a qualitative content analysis, completed by a discourse analysis taking into account the context of the IDK-Qs (cotext), their intent and their content; content was largely coded using the RIAS categorization [35,36].

This study was approved by the Ethics Committee of the University Hospital of Lausanne.

## Results

### Frequency of IDK-Qs

Twenty-six of the 63 (41.3%) students asked 1-6 IDK-Qs during the interviews. In total, 53 IDK-Qs occurred, of which 49 had a rising pitch.

No significant gender or scenario effect was observed: female (N = 38) and male (N = 25) students used IDK-Qs in almost equal proportions (39.5% vs. 44%, *p = 0.995*) and the ratio of IDK-Qs in the interviews with the curative scenario (N = 33) was higher than within the palliative scenario (N = 30) (67.9% vs. 32.1%, *p = *0.127).

### Content of IDK-Qs

Based on their content, IDK-Qs were attributed to three broad categories (see Table 1): medical/treatment questions (N = 24), e.g. *I don't know if you have already heard about chemotherapies *[S53]; lifestyle/psychosocial questions (N = 18), e.g. *I don't know if you have any, some plans for, for the future *[S6]; and "inviting questions" questions (N = 11), e.g. *I don't know if you have other questions *[S51] (each IDK-Q is presented with its interview number and author (S = Student, O = Oncologist, SP = Simulated Patient).

**Table 1:** Content of IDK-Qs

Content categories	
Medical/treatment questions (N = 24)	*I don't know if you know more or less of what it consists or not at all [chemotherapy and radiotherapy] *[S11] - RP, FS, CS *I don't know if you have any, any a priori, if you have heard about, about chemotherapy *[S13] - FP, FS, CS *I don't know if your attending physician has explained you that it would be more difficult to treat [if the ganglia were affected] [S14] - RP, FS, CS *I don't know if you have also other questions concerning this matter [the treatment] *[S23] - RP, MS, CS *I don't know if you know a bit of [chemotherapy], if one has already talked to you *[S24] - RP, FS, CS *I don't know if you knew [about the prognosis] [S28] - RP, FS, PS *I don't know what questions do you have about this [the proposed treatment] *[S28] - RP, FS, PS *I don't know if you know what this is [a CT (scan)] [S28] - RP, FS, PS *I don't know if you know why we called you *[S31] - RP, MS, PS *I don't know if you know what this means [immunodepressed] [S34] - RP, FS, CS *I don't know if you have already heard about these words [chemotherapy and radiotherapy], if you know what this means *[S34] - RP, FS, CS *I don't know if you have any questions about what comes next or about the chemotherapy treatment *[S41] - FP, FS, PS *I don't know if you have any questions about that [chemotherapy and radiotherapy] *[S43] - RP, FS, CS *I don't know if you have any questions about these two treatments [chemotherapy and radiotherapy] *[S44] - RP, MS, CS *I don't know if you have any questions [about chemotherapy and radiotherapy] *[S45] - RP, MS, CS *I don't know if maybe you don't want to speak about or to the contrary you want to speak about [the side effects of chemotherapy] *[S45] - RP, MS, CS *I don't know if you have already heard about [radiotherapy] *[S49] - RP, MS, CS *I don't know if for you it, it suits you as treatment *[S49] - RP, MS, CS *I don't know if you want me to talk to you about it [chemotherapy side effects] *[S50] - RP, MS, CS *I don't know if you have already heard about chemotherapies *[S53] - FP, FS, CS *I don't know if you knew [that the treatment has to be pursued] [S53] - RP, FS, CS *I don't know how your are feeling today after this surgery *[S57] - RP, MS, CS *I don't know if you want to talk about it today [therapeutic means] or if you rather want to take time to think about it at home *[S61] - RP, FS, PS *I don't know if you already know a bit what it is [chemotherapy] *[S63] - RP, MS, PS

Lifestyle/psychosocial questions (N = 18)	*I don't know if, if you want to come to the next appointment with one of your relatives, who, who can support you or if you want to come alone *[S2] - RP, FS, PS *I don't know if you are an employer *[S2] - RP, FS, PS *I don't know if you have any, some plans for, for the future *[S6] - RP, MS, PS *I don't know if your *(family/friend) *circle if they know what you have or *[S11] - RP, FS, CS *I don't know if you work [S11] - RP, FS, CS *I don't know what you prefer to do in what follows *[S11] - RP, FS, CS *I don't know concerning your son what you want to do *[S11] - RP, FS, CS *I don't know actually you how you like that it happens *[S24] - RP, FS, CS *I don't know how you feel *[S28] - RP, FS, PS *I don't know if you work or [S35] - RP, FS, CS *I don't know what kind of job you have, what you are *[S36] - RP, FS, CS *I don't know if you have special enough relationships with your family doctor or with someone who knows a bit of your situation *[S39] - RP, MS, PS *I don't know you how you feel *[S44] - RP, MS, CS *I don't know what you do (for a living) *[S44] - RP, MS, CS *I don't know if you want him to come [the patient's son] to the next meeting, to one of the next sessions or if rather not *[S45] - RP, MS, CS *I don't know if you would like to, I mean, to make an appointment with your relatives as well such that we can talk about it all together *[S53] - FP, FS, CS *I don't know if you prefer [that the nursing staff explains the situation to the patient's children] or maybe come to the next appointment with your family *[S61] - RP, FS, PS *I don't know if for you it's awkward [the hair loss] *[S63] - RP, MS, PS

"Inviting questions" questions (N = 11)	*I don't know if you have any questions about, about anything, how, how it will happen *[S6] - RP, MS, PS *I don't know if you have already questions now *[S11] - RP, FS, CS *I don't know if you have any questions *[S44] - RP, MS, CS *I don't know if you have other questions that come to mind *[S45] - RP, MS, CS *I don't know if you have other questions, other things to ask me or other worries *[S46] - RP, FS, CS *I don't know if you have also a precise question now *[S50] - RP, MS, CS *I don't know if you have other questions *[S51] - RP, MS, PS *I don't know if you have until now any questions *[S53] - RP, FS, CS *I don't know if you have any questions *[S53] - RP, FS, CS *I don't know if you have already like that if you have something to ask me *[S59] - RP, FS, CS *I don't know if you have other questions *[S61] - RP, FS, PS

* IDK-Qs with precise information request

**RP**/**FP**: Rising Pitch/Falling Pitch

**FS**/**MS **female student/male student

**CS**/**PS **curative scenario/palliative scenario

The medical/treatment category included IDK-Qs about medical anamnesis, medical condition, symptoms, diagnosis, prognosis, treatment, side effects, as well as IDK-Qs assessing patient's perception of the medical situation and knowledge of anticancer treatments and inviting questions about the treatment. IDK-Qs in the lifestyle/psychosocial category addressed family and home situation, patient's employment, support, state of mind, psychological concerns and needs, and involvement of patient's relatives. Finally, the category of IDK-Qs "inviting questions" included broad probes for questions or information about unspecified topics.

### Context of occurrence of IDK-Qs

IDK-Qs about medical and treatment issues mainly occurred in the first stages of the interviews after introducing one selves, students' probes about the reason for the consultation (opening stage) and the disclosure of diagnosis or treatment options. IDK-Qs about lifestyle and psychosocial aspects tended to be asked after discussion of treatment options and medical issues, while IDK-Qs inviting questions occurred at all stages of the interviews.

### Intent function of IDK-Qs

#### Precise information requests (N = 7)

Seven IDK-Qs were classified as specific (indirect) questions since their seeming function was to request precise information: e.g., *I don't know if you work *[S11], *I don't know if you knew [about the prognosis] *[S28], *I don't know if you know what this means [immunodepressed] *[S34] (all IDK-Qs with precise information requests are marked by an * in Table 1).

In reply to these questions, the simulated patients provided minimal information (e.g., *not really *[SP28], non-verbal *no *[SP34]), probably interpreting these IDK-Qs as a request for clarification.

#### Exploratory requests (N = 46)

In addition to IDK-Qs inviting questions, which have a large scope and generally (7/11) led to extended responses or requests for more information (e.g., *I don't know if you have already questions now *[S11]), 35 IDK-Qs seemed to have an exploratory function.

In discussions about the treatment plan (medical/treatment IDK-Qs), IDK-Qs were intended, for example, to find out what the (simulated) patient already knows (*I don't know if you know a bit of [chemotherapy], if one has already talked to you *[S24]), if he/she has any queries about the treatment (*I don't know if you have any questions about these two treatments [chemotherapy and radiotherapy] *[S44]) or if he/she wants to be informed about the side effects (*I don't know if maybe you don't want to speak about or to the contrary you want to speak about [the side effects of chemotherapy] *[S45]).

Concerning the psychosocial aspects (lifestyle/psychosocial IDK-Qs), IDK-Qs aimed, for example, to explore social support (*I don't know if you have special enough relationships with your family doctor or with someone who knows a bit of your situation *[S39]), involvement of relatives in the treatment process (*I don't know if you would like to, I mean, to make an appointment with your relatives as well such that we can talk about it all together *[S53]) or to disclose patient's concerns related to the future or to distressing side effects of chemotherapy [*I don't know if for you it's awkward [the hair loss] *[S63]).

Twenty-nine of these "scanning" questions (29/35) elicited extensive responses and six short or yes/no responses (6/35).

### Simulated patient replies to IDK-Qs

In reply to 36 of the 53 IDK-Qs, simulated patients provided detailed responses and/or asked for more information. The fact that some IDK-Qs (N = 4) were pronounced with a falling pitch apparently didn't affect the responses, since simulated patients continued with extended information.

The following three contextualized excerpts of interviews illustrated the type of extended responses or requests for more information formulated by the simulated patients and at the same time shed some light on the interaction dynamics surrounding the IDK-Qs.

In excerpt 1, the actress simulating a breast cancer patient (SP) responded to an IDK-Q of a student (S) inviting questions in the "summary stage" of the interview (at 13 out of 15 min):

S45: I don't know if you have other questions that come to mind.

SP45: (2 s) Regarding my son, because I have a seven-year-old son, it's true that (1 s) we haven't really said to him what happened. So, I don't know whether (2 s) what I'm supposed to do for him, whether I'm supposed to tell him or not.

S45: (6 s) That's, I think that's really a personal choice, that's (1 s) it's true that he will see his mum tired, that he will see his mum without [hair?], who often has to go to the doctor, maybe you could try to explain to him. I think that will be hard.

(The student then asked another IDK-Q: I don't know if you want him to come to the next meeting, to one of the next sessions or if rather not.)

In excerpt 2, the student's IDK-Q about chemotherapy occurred at the beginning of the interview (at 2 min) after the disclosure of the breast cancer diagnosis and treatment options:

S53: I don't know if you have already heard about chemotherapies.

SP53: I think that the side effects are still quite serious. At least as far as I know.

S53: Mm-hm.

SP53: Regarding tiredness, (1 s) hair loss, all this stuff.

S53: Okay (the student started to explain the side effects mentioned by the simulated patient.)

Excerpt 3 occurred in the first third of the interview (at 8 min). After the student has informed about the diagnosis of stomach cancer, addressed patient's concerns and disclosed the (poor) prognosis, she asked by an IDK-Q how the simulated patient felt (psychologically):

S28: I don't know how you feel.

SP28: I don't feel me anymore. I don't feel anything.

S28: Mm-hm.

SP28: It's emptiness, it's a black hole that pulls me in. It's, even no desire to scream, (1 s) I can't have a grudge against anybody.

S28: Mm-hm.

SP28: The doctor, the surgeon has done what he has done.

S28: Mm-hm.

SP28: I'm (6 s) I can't fall asleep to forget, that's all (4 s) I understand those who jump from a bridge.

S28: (8 s) You told me earlier your short-term projects, you already talked about death and dying, that you will ask your relatives to incinerate you in a place you like and that you want to settle your affairs somewhat. After this news, is that still (1 s) something that you are planning to do?

### Comparison with the oncologists

Five IDK-Qs were identified in the interviews of the 31 oncologists: three oncologists formulating 1 IDK-Q and one oncologist formulating 2 IDK-Qs. With regard to their content, three IDK-Qs concerned medical/treatment issues - *I don't know if one has already talked to you about side effects *[O1165], *I don't know how you, you see the stuff chemotherapy, what does it mean for you *[O1209], *I don't know if there were any breast cancers in your family *[O1240] - and two lifestyle/psychosocial issues - *I don't know if you are married, if you have children *[O1240], *I don't know what it means for you to, to think of, of losing the hair *[O1266]. Except for the one about the medical history of the family, the IDK-Qs appeared to have an exploratory intent; simulated patients provided extensive responses in relation to the hair loss [SP1266] and to their representation of chemotherapy [SP1209].

The oncologist IDK-Qs corresponded to the same content categories - except for the "inviting questions" category - and mainly functioned as exploratory questions, but they were rare compared to the student sample (5 vs. 53 occurrences, 4/31 oncologists vs. 26/63 students).

## Discussion

IDK-Qs were a distinguishing feature of the student (compared to oncologist) discourse, which suggests at first sight, with regard to the hypotheses of the study, a sociolinguistic explanation. The use of IDK-Qs could be a generational marker and expression of a youth language spoken by certain students. It could also indicate that students and oncologists belong to different social groups with specific language habits or praxis (sociolectal marker).

However, the content of the IDK-Qs, the information they targeted and the fact that they did not represent the common manner of asking questions (otherwise the number of their occurrences would be much higher in the interviews) call for further psychological explanations.

First, the majority of the student IDK-Qs were generally used to initiate discussion on treatment options and medical issues. This is the core of breaking bad news [11,12] and students had to find out where the (simulated) patient stands in relation to his/her disease and to cope with his/her possible negative representations concerning the treatment. Since investigating these matters is anxiety-provoking, IDK-Qs could be understood as a defensive attitude of the students to tone down the impact of their questions and to avoid distressing the (simulated) patients and themselves. IDK-Qs would therefore represent a marker for student inexperience with these matters leading to insecurity and discomfort.

Second, student lifestyle/psychosocial IDK-Qs aimed to explore (simulated) patient's concerns, emotions and social support, to involve relatives and to examine professional commitments. Based on our observations with this course on breaking bad news, students might also feel uncomfortable when addressing psychosocial issues, since they tend to avoid them. They probably considered an IDK-Q like *I don't know if you have some plans for the future *as less intrusive and less confronting than a direct question like *have you some plans for the future?*

Third, the students used IDK-Qs for inviting questions, i.e., asking what the patient wants to know. Asking what the patient wants to know in a breaking bad news setting gives the patient space to ask questions or to request more information. It is quite probable that students feel lacking in the necessary experience to respond to (simulated) patient questions, and consequently that these IDK-Qs are a marker for insecurity in providing space to simulated patients.

The psychological explanation of IDK-Qs in the student discourse implies that oncologists - who rarely utilized IDK-Qs - might be less anxious when conducting simulated patient interviews or that they have other protective mechanisms. Studies examining oncology clinician defense mechanisms in the context of CST [38,39] have shown that a great number and variety of defense mechanisms are triggered by simulated patient interviews, indicating a high level of stress; the most frequently observed defense mechanisms were, for example, *intellectualization *(using medical or technical explanations in response to the patient emotional distress) and *rationalization *(disavowing the emotional experience of the patient by "reassuring" statements). In other words, oncologists might use defense mechanisms that relate to their medical knowledge to cope with anxiety, while students do not have this medical background and therefore use IDK-Qs to protect themselves.

From an educational point of view, IDK-Qs may allow to focus the training on student needs by identifying issues that make them feel uneasy and potentially provoke anxiety.

In most cases the simulated patients responded extensively to IDK-Qs, which suggests that they did not have a blocking effect. It might be that IDK-Qs represent a self-protection communication behavior of the students, which is unlikely to be perceived as such by the simulated patients, that does not block the verbal expression of the simulated patients. It might also be that IDK-Qs mirror student awareness - due to communication skills teaching during all undergraduate years - of the psychological processes and communication challenges involved in breaking bad news and show thereby the attention they pay to the (simulated) patients by cautiously exploring sensitive topics. Oncologists, on the other hand, might rarely use IDK-Qs because of their less substantial training in communication skills. IDK-Qs would then provide some information about differences of patient-centered communication style between students and oncologists. For their part, simulated patients might interpret IDK-Qs as a way to center communication on them and consequently as an invitation to speak. However, it is also possible that simulated patients just seized the vagueness of indirect questioning as an opportunity to express themselves. Finally, IDK-Qs could reflect a non- or less dominant attitude of the students - congruent with their inexperienced status - inducing narrativity in the simulated patients.

The main limitation of this exploratory study was that it addressed one specific format of questions regardless of other questions asked by medical students in simulated patient interviews. In spite of their recurrence, IDK-Qs were not the most frequently asked questions and most probably not the only kind of indirect questioning used by students. However that may be, it seems that IDK-Qs throw some light both on how students actually interact with patients in simulated breaking bad news interviews and on the communication skills of Master medical students.

## Conclusions

It might be worthwhile to expand this study with different samples and settings (including interviews with actual patients) in order to determine the signification(s) of IDK-Qs and confirm their possibly lessened blocking effect on (simulated) patients. In addition, other types of indirect questioning could be taken into consideration to obtain a contrastive point of view. If the hypothesis would be further confirmed that IDK-Qs can be considered as a protective linguistic strategy and a marker for psychological discomfort, they could be used for feedback and analysis of videotaped interviews in CST or for scientific purposes (e.g., as an easily identifiable indicator for evaluation of CST outcomes).

## Competing interests

The authors declare that they have no competing interests.

## Authors' contributions

CB was the principal investigator. FS and AB contributed to the acquisition of data. CB, FS, AB and PS contributed to the conception and design of the exploratory study. CB and FS were involved in interpreting the data and drafted the article. All authors critically revised the article and approved the final manuscript.

## Pre-publication history

The pre-publication history for this paper can be accessed here:

http://www.biomedcentral.com/1472-6920/12/8/prepub
